# A comparative analysis of adverse drug reactions: pre- and post regional pharmacovigilance center in a single tertiary hospital

**DOI:** 10.1186/1939-4551-8-S1-A60

**Published:** 2015-04-08

**Authors:** Young-Hee Nam, Hee-Joo Nam, Jeong-Eun Song, Soo-Keol Lee

**Affiliations:** 1 Dong-a University School of Medicine, South Korea; 2 Department of Pharmacy, Dong-a University Hospital, Sourth Korea; 3 Dong-a University Hospital Regional Pharmacovigilance Center, South Korea

## Background

The number of adverse drug reactions (ADRs) has been significantly increased since the introduction of regional pharmacovigilance centers (RPVC) in Korea. We compared the reporting and clinical features of ADRs after starting the RPVC in a single tertiary hospital, and investigated the attitude and knowledge of ADR reporting and pharmacovigilance.

## Methods

ADR data were collected from April 2012 to November 2013 in Dong-A university hospital in which started the RPVC since February 2013. We compared the ADR data before and after starting the RPVC. A questionnaire survey was conducted in a total of 436 health care workers.

## Results

The total number of reported ADRs increased from 420 to 1265. The most common ADR reporters were doctors (59.4%), followed by nurses (29.4%) which was significantly increased from 2012 (8.8%). The most common causative drugs were antibiotics (30.6%), followed by miscellaneous drugs (30.4%), and antituberculosis drugs (10%), and clinical manifestations of ADRs were cutaneous (41.9%), gastrointestinal (24.9%) and neurophsychiatric symptoms (18.5). The distribution of causative drugs and clinical manifestations were differed from those in 2012. Majority of the health care workers reported ADR reporting is necessary (98.9%) and is their obligation (96.3%), while only 38.1% have reported ADR.

## Conclusions

The number of reported ADRs significantly increased since the introduction of RPVC in a tertiary hospital. The causative drugs and clinical manifestations of ADRs changed. The ADR reporters diversified and they have the knowledge and responsibility to ADR reporting.

